# Influence of Insect Growth Regulators on *Stephanitis pyrioides* (Hemiptera: Tingidae) Eggs and Nymphs

**DOI:** 10.3390/insects10070189

**Published:** 2019-06-28

**Authors:** Shimat V. Joseph

**Affiliations:** Department of Entomology, University of Georgia, UGA Griffin Campus, 1109 Experiment Street, Griffin GA 30223, USA; svjoseph@uga.edu; Tel.: +1-770-228-7312

**Keywords:** insect growth regulator, novaluron, azalea lace bug, azalea, ovicide

## Abstract

The azalea lace bug, *Stephanitis pyrioides* (Scott) (Hemiptera: Tingidae), is an important insect pest of azaleas (*Rhododendron* L. spp.) in the USA. *Stephanitis pyrioides* feeds on azalea foliage and causes extensive chlorosis, which reduces the aesthetic value and marketability of these plants. Because the use of neonicotinoid insecticides has been dramatically reduced or discontinued, growers and landscape managers are seeking alternative tools or strategies to control this insect. Although insect growth regulators (IGRs) are known for their activity against immature insect stages, their activity against egg hatching has not been addressed thoroughly, specifically against *S. pyrioides*. Thus, a series of experiments was conducted to understand the ovicidal activity of IGRs using novaluron, azadirachtin, pyriproxyfen, and buprofezin against *S. pyrioides*. The number of newly emerged young instars was significantly lower when leaves implanted with eggs were sprayed on both sides with novaluron, azadirachtin, and buprofezin compared to nontreated and pyriproxyfen treatments. When IGRs plus adjuvant were applied to the adaxial surface of the leaves, the densities of the newly emerged nymphs were significantly lower under the novaluron treatment compared to the nontreated leaves. However, there was no significant difference in the number of nymphs that emerged in the absence of adjuvant. Furthermore, close monitoring revealed reduced levels of egg hatching in the presence of adjuvant with novaluron compared to its absence. The data show that the survival of *S. pyrioides* first instars was not affected by exposure to dried IGR residues.

## 1. Introduction

The azalea lace bug, *Stephanitis pyrioides* (Scott) (Hemiptera: Tingidae), is an important insect pest of azalea plants (*Rhododendron* L. spp; family Ericaceae) in the eastern USA [1]. In recent years, *S. pyrioides* has become established in Oregon and Washington, and it poses a serious threat to *Rhododendron* L. spp in production nurseries and landscapes [2,3]. The affected leaves develop yellow speckles, or in extreme cases, they will appear completely bleached or chlorotic because both the nymphs and adults of *S. pyrioides* feed on chlorophyll [4,5,6]. In the ornamental nursery industry, *S. pyrioides*-infested plants cannot be marketed, and infestations of public and private landscapes or gardens reduces their aesthetic value [4,5]. 

Until recently, *S. pyrioides* infestations in nurseries, as well as landscapes, have been managed using the effective neonicotinoid insecticide, imidacloprid [7]. In early April, a granular formulation of imidacloprid is applied, and it provides year-long pest control. However, neonicotinoid insecticide use in private and public landscapes is perceived as a threat to pollinators and other beneficial arthropods. Therefore, the market demand for neonicotinoid-treated nursery plants has declined. Similarly, in landscapes, neonicotinoid insecticide use on established azalea bushes for *S. pyrioides* management has dropped. At present, nursery growers and landscape managers resort to multiple sprays of pyrethroid insecticides, which can have negative impacts on beneficial arthropods and increase the risk of secondary pest outbreaks [8]. Thus, the ornamental industry in the eastern USA is seeking alternative, cost-effective, and sustainable options for *S. pyrioides* control. 

In the field, *S. pyrioides* eggs are implanted into leaf tissue along either side of the midrib and lateral veins on the abaxial surfaces of azalea leaves [1]. Once the eggs are inserted, the female *S. pyrioides* deposits cement-like fecal matter on the operculum of the eggs, and thus, the eggs are mostly hidden and protected [6]. Occasionally, the oval-shaped opercula of the eggs are visible on the leaf surface. In the eastern USA, *S. pyrioides* overwinters as eggs, and the eggs hatch starting in March [9,10]. The early season management of these overwintering eggs or emerging young nymphs can considerably reduce population buildups of *S. pyrioides* later in the season. 

Insect growth regulators (IGRs) are regarded as reduced-risk insecticides because of their low toxicity to nontarget organisms, especially mammals, and they are known to target immature stages of insect pests [11]. Previous studies have shown that IGRs elicit transovarial activity in the adult *S. pyrioides* when only the adults are directly and indirectly exposed to dried IGR residues [12]. These IGRs were not evaluated for their ovicidal or nymphicidal activities. The IGRs that are effective as ovicides or nymphicides can play a critical role in managing the *S. pyrioides* population development on azalea plants because developing nymphs also feed on azalea foliage and can cause substantial aesthetic damage. Evidence of ovicidal activity has been shown on other hemipterans such as the tarnished plant bug, *Lygus lineolaris* (Palisot de Beauvois) [13]. In the current study, four IGR insecticides, novaluron, azadirachtin, pyriproxyfen, and buprofezin, were evaluated for ovicidal and nymphicidal activity against *S. pyrioides*. Novaluron, a benzoylurea insecticide, is widely registered for the management of major agricultural and ornamental pests. Novaluron is classified as a chitin biosynthesis inhibitor (Insecticide Resistance Action Committee, Group 15) [14] because it disrupts the biosynthesis of the insect cuticle [15,16,17]. Another common IGR, azadirachtin, is a tetranortriterpenoid insecticide that is derived from the seed oils of the neem tree (*Azadirachta indica* A. Juss.). Although the exact mode of action of azadirachtin is not well understood (Insecticide Resistance Action Committee, Group UN) [14], it is known to alter the biosynthesis of the insect hormone ecdysone and inhibits insect molting. The pyridine-based insecticide pyriproxyfen is widely used against piercing and sucking pests [18,19]. Pyriproxyfen is a juvenile hormone analog (Insecticide Resistance Action Committee, Group 7C) [14,18,19] that affects insect molting. The thiodiazin derivative insecticide buprofezin is known to be effective against piercing and sucking pests [20,21,22,23] because it affects insect molting by inhibiting chitin biosynthesis (Insecticide Resistance Action Committee, Group 16) [14].

The objective of this study was to determine the activities of novaluron, azadirachtin, pyriproxyfen, and buprofezin against *S. pyrioides* eggs and nymphs by directly exposing them to dried IGR residues or to IGRs via translaminar movement. If one or more IGRs affect *S. pyrioides* egg hatching and/or nymph survival, early applications of the best IGR could be targeted towards overwintering eggs and the first generation of young nymphs to suppress their population buildup later in the season. 

## 2. Materials and Methods

### 2.1. Plants and Insects

A *S. pyrioides* colony was maintained at the University of Georgia’s entomological laboratory on live ‘George Tabor’ azalea plants in 3.7 L pots, in cages, in Griffin, Georgia, USA. These plants served as a food and water source for the nymphs and adults as well as the oviposition substrate of the *S. pyrioides* adults. A fresh plant was introduced at six-week intervals to ensure a continuous supply of food and water for the *S. pyrioides*. These plants were not exposed to insecticides or other chemicals. For experiments related to the topical spraying of implanted eggs, live ‘Pink Ruffle’ azalea plants in 3.7 L pots were used, and the remaining trials were conducted on ‘George Tabor’ azalea plants in 3.7 L pots. Both cultivars are equally susceptible to *S. pyrioides* infestations and damage. *Stephanitis pyrioides* adults were initially collected from azalea shrubs that displayed a natural infestation in Griffin, Georgia. The caged *S. pyrioides* host plants were placed on laboratory racks under ~55% relative humidity at ~39 °C – ~22 °C (day:night) and a 16:8 h (light:dark) photoperiod. These caged plants were placed under incandescent lamps (Philips, 40 W, Andover, MA, USA), which served as heat as well as light sources. *Stephanitis pyrioides* completed a life cycle within approximately 1 month under these laboratory conditions. Adults aged 6 and 7 d old were used for various assays.

### 2.2. Insecticides

The IGRs used in the assays were novaluron (Pedestal^®^ (10% a.i.), OHP Inc., Bluffton, SC, USA), azadirachtin (Azatin-O^®^ (4.5% a.i.) Botanical Insecticide, OHP Inc., Bluffton, SC, USA), pyriproxyfen (Fulcrum^®^ (11.2% a.i.), OHP Inc., Bluffton, SC, USA), and buprofezin (Talus 70DF^®^ (70% a.i.), SePRO corporation, Carmel, IN, USA). The rates of the active ingredients used in all the assays were 58.1, 54.6, 90.3, and 685.9 g per ha for novaluron, azadirachtin, pyriproxyfen, and buprofezin, respectively. Because the water volume in use generally varied between 280.6 and 560.7 L per ha during nursery production, an intermediate water volume of 373.9 L per ha was selected to determine the insecticide concentration for the assays. Thus, the concentrations of active ingredients in the solutions were 155.4, 145.9, 241.7, and 1834.2 ppm for novaluron, azadirachtin, pyriproxyfen, and buprofezin, respectively. These insecticide rates were determined based on either labelled for azalea or *S. pyrioides* or registered on a closely related crop or pest. For some experiments (as indicated in the following sections), a nonionic surfactant (adjuvant), (Dyne-Amic^®^, Helena Agrichemicals, Collierville, TN, USA) (99% of methyl esters of C16-C18 fatty acids, polyalkyleneoxide modified polydimethylsiloxane, and alkylphenol ethoxylate,) was added to the IGR treatments at 0.25% *v*/*v*. 

### 2.3. Topical Spray on Implanted Eggs

This experiment was conducted on ‘Pink Ruffle’ azalea plants in 3.7 L pots. *Stephanitis pyrioides* adults aged 6 and 7 d old are typically sexually mature and mated. For the assay, 10 adults were randomly collected from the rearing colony and used in the assay. Although these adults were not separated by gender when they were collected from the cages, the populations in the rearing cages had a 1:1 sex ratio. The collected adults were caged on the terminal branch of a potted azalea plant with 10–14 mature leaves using a 14 × 11 cm sleeve mesh cage (length:width) for 14 d. This is a sufficient period for the *S. pyrioides* females to implant their eggs into the azalea leaves. After 14 d, the adults were removed using hand-held aspirators from previously caged branches, and the insecticide solutions were spray-applied to the abaxial and adaxial surfaces of the leaves using a hand-operated pressure sprayer. The end of the cage was secured to the azalea stem by pulling the cage’s strings. The branches were caged again to exclude any *S. pyrioides* reinfestation. 

Novaluron, azadirachtin, pyriproxyfen, buprofezin, and the water control made up the treatments. The treatments were replicated five times in a randomized complete block design in which each individually caged terminal was the replicate. The treatments were blocked by the azalea plants in which each plant received all the treatments. The caged, potted plants were placed on a laboratory rack (~30 °C (day), ~20 °C (night), 20–40% relative humidity, and 16:8 h (light:dark) photoperiod), and incandescent lights (Philips, 40 W) were directed at them. 

After 7 d of insecticide application, the azalea branches were removed destructively for evaluation. The leaves were stripped from the branches and thoroughly examined for the presence of nymphs under a dissecting microscope. The nymphs observed on the leaf samples were separated into first, second, third and fourth, and fifth instar stages. This experiment was repeated four times. For the first two repeats, the nymphs were not separated by instars, but for the last two repeats, the nymphs were separated into their various nymphal stages. The nymphs shed their old exoskeleton (exuviae) when they molt, and the number of exuviae per assay were quantified. The exuviae is translucent or white in color. The trials were initiated on June 11, July 2 and November 5, 2018, and January 18, 2019 for trials 1, 2, 3, and 4, respectively. The assays were evaluated on July 2, 23, and November 26, 2018; and February 8, 2019 for trials 1, 2, 3, and 4, respectively. 

### 2.4. Translaminar Effects on Implanted Eggs and Nymphs

This experiment was conducted on ‘George Tabor’ azalea plants in 3.7 L pots. From the colony, 10 *S. pyrioides* adults were randomly collected and caged on a terminal branch with 10–15 mature leaves for 14 d. This is a sufficient period for *S. pyrioides* females to implant their eggs into the azalea leaves. After 14 d, the adults were removed from the individually caged branches. The adaxial side of the leaves in the cages were gently painted with the insecticide solution using a paint brush. Novaluron, azadirachtin, pyriproxyfen, buprofezin, and the water control were the treatments. The exposed terminal branches were caged again for 7 d to avoid any reinfestation by *S. pyrioides*. 

Two types of experiments were conducted in which (1) an adjuvant was not added and (2) an adjuvant (Dyne-Amic^®^) was added at 0.25% *v*/*v* to all the IGR treatments. Each assay was arranged in a randomized complete block design with five replications. Each terminal branch served as an experimental unit. All the treatments were blocked by assigning one to each potted azalea plant. The adults used in the experiments were ~7 d old. The caged potted plants were placed on a laboratory rack (~30 °C (day), ~20 °C (night), 20–40% relative humidity, and 16:8 h (light: dark) photoperiod), and incandescent lights (Philips, 40 W) were directed onto the plants. 

After 7 d of being painted with insecticide, the azalea branches were removed and their leaves were thoroughly examined for *S. pyrioides* nymphs. The nymphs observed on the leaf samples were separated into first, second, third and fourth, and fifth instar stages. The shed skin and number of leaves per assay were also quantified. The experiments were repeated three times per type (with or without adjuvant). The trials without adjuvant were initiated on November 6 and 9, 2018; and February 18, 2019 for trials 1, 2, and 3, respectively. The assays were evaluated on November 27 and 30, 2018; and March 11, 2019 for trials 1, 2, and 3, respectively. The trials with adjuvant were initiated on November 19 and 27, 2018; and February 8, 2019 for trials 1, 2, and 3, respectively. The assays were evaluated on December 10 and 18, 2018; and March 1, 2019 for trials 1, 2, and 3, respectively.

### 2.5. Assessing Ovicidal Effects

A study was conducted to understand the effects of novaluron on egg viability. Only novaluron was chosen for this assay because it appeared to affect the nymph production number during the preliminary assays. Six *S*. *pyrioides* adults were caged on a 10-cm long, ‘George Tabor’ azalea terminal branch with two mature leaves for 7 d. This terminal branch was placed in a water-filled, translucent polypropylene cup (6 cm diam. wide and 7.1 cm long). A 5-mm hole was drilled on the center of the lid of the translucent container and the stem of the azalea terminal was inserted through the hole to keep the leaves alive. The container containing the azalea branch was placed inside a 2-L clear plastic container with a clear lid. A water-soaked paper was placed inside the clear container to increase the relative humidity inside the clear container so the *S. pyrioides* adults and eggs would survive. These clear 2-L containers were placed on a laboratory rack (~30 °C (day), ~20 °C (night), 90% relative humidity, and 16:8 h (light:dark) photoperiod), and incandescent lights (Philips, 40 W) were directed at them. After 7 d, the adults were removed using aspirators. The leaves containing implanted eggs were dipped in novaluron solution or water for 15 s and then placed on dry tissue paper for 15 min. After 15 min, the terminals were reintroduced into the cup and placed on the 2-L clear containers. Because the *S. pyrioides* females deposited black cement-like material on the eggs, the accurate egg densities on the leaves could not be viewed through a stereomicroscope and quantified. Thus, the numbers of eggs on the leaves were not quantified. Thereafter, the nymphal eclosion was recorded every day for up to 15 d under the stereomicroscope. Whenever newly hatched first instars were found on the leaf surface, they were removed using a needle. 

Two types of experiments were conducted involving (1) novaluron without an adjuvant and (2) novaluron with an adjuvant (Dyne-Amic^®^) at 0.25% *v*/*v*. Each experiment type (without and with adjuvant) had five replications per treatment (novaluron and a nontreated water control) in a completely randomized design on the laboratory rack. The individual terminal branch was one replication. Each experimental type was repeated three times. The trials were initiated on March 26 and 28, and April 22, 2019 for trials 1, 2 and 3, respectively. These trials were evaluated daily for up to 15 d. Preliminary assays showed that the adjuvant by itself had no effects on egg hatching.

### 2.6. Direct Exposure on First Instars

Individually potted ‘George Tabor’ azalea plants were treated with novaluron, azadirachtin, pyriproxyfen, buprofezin and a water control. The treated potted plants were placed on a laboratory rack (~30 °C (day), ~20 °C (night), 20–40% relative humidity, and 16:8 h (light: dark) photoperiod), and incandescent lights (Philips, 40 W) were directed towards them. The mature leaves were sampled from these treated plants and placed in Petri dishes individually. To set up the assay, five first instars were carefully transferred onto the treated leaves (various IGRs or water). Once the transfer was completed, the Petri dishes were sealed using Parafilm on the sides to prevent moisture loss. 

This Petri dish assay was set up two times using IGR-treated leaves, at 1 and 7 d after the IGRs were spray-applied to the potted plants. For each assay (1 or 7 d), the leaves treated with various IGRs were evaluated at 3 and 24 h after their introduction to a record number of live first instars per leaf after a specific period. The treatments consisted of IGRs and the water control, and they were replicated five times (five dishes) in a completely randomized design on the laboratory bench. The entire experiment was repeated two times. The trials were initiated on November 29, 2018 and January 9, 2019 for trials 1 and 2, respectively. The trial 1 assays were evaluated on November 29 and 30 as well as December 6 and 7, 2018, whereas the trial 2 assays were evaluated on January 9 and 10 and 16 and 17, 2019.

### 2.7. Statistical Analysis

All the statistical analyses on the data were performed in SAS [24]. The data from all the repeated trials for each experiment types were combined because the individual experiments or assays were repeated and evaluated using exactly the same protocol. For the topical spray onto the implanted eggs, because various *S. pyrioides* instars were identified and recorded, trials 3 and 4 were combined to determine the effects of the treatments on the development of specific *S. pyrioides* instars before statistical analysis. However, when the overall effect of the IGRs on all the nymphal stages were combined and the exuviae was sought, all four repeats were combined before the statistical analysis. Similarly, the data from the repeated trials from the translaminar experiments and the experiments to assess the ovicidal effects were combined by experiment types (with and without adjuvant). For the data obtained from the topical spray and translaminar movement, an ANOVA was performed in PROC GLIMMIX in SAS during which the IGR treatments and replications were assigned as fixed and random effects, respectively, in the model. These analyses had a log link function and negative binomial distribution. The least squares means were separated by pairwise *t*-test (*p* < 0.05). 

To determine the effect of novaluron (with or without adjuvant) on nymphal eclosion, the first instars that emerged from three repeats per experimental type were combined and log-transformed (ln[x + 1]), and paired Student’s *t*-test analysis was performed using the PROC TTEST procedure in SAS at *α* = 0.05. The novaluron-treated and untreated samples made up the treatments. The means and standard errors of the variables were calculated using the PROC MEANS procedure in SAS. To determine the effects of direct IGR exposure on the first instars of *S. pyrioides*, the data from two trials were combined according to the age of the residue (1 or 7 d), and the data were subjected to ANOVA using a general linear model (PROC GLM) in which the IGR treatment, exposure time (3 and 24 h) and treatment × exposure time interaction were addressed. A separate analysis was conducted for each residue age (1 and 7 d). Furthermore, one-way ANOVAs were performed by exposure time (3 and 24 h) to determine the effect of the given IGR treatment on the mortality of the first *S. pyrioides* instars. The means were separated using Tukey’s HSD test for treatment comparisons. All the statistical comparisons were considered significant at α = 0.05.

## 3. Results

### 3.1. Topical Spray on Implanted Eggs

**Figure 1:**
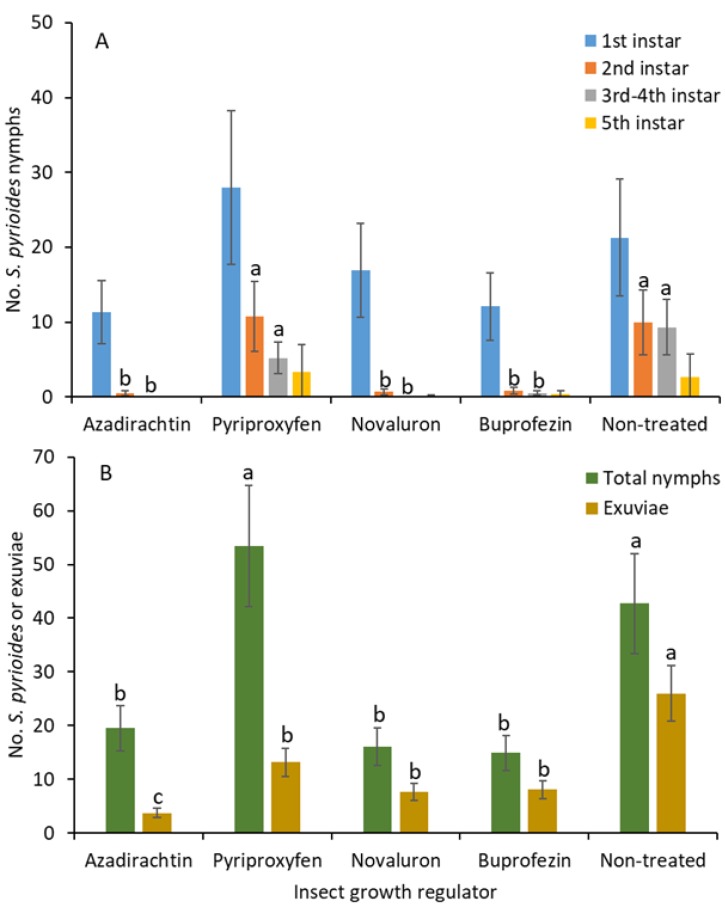
Least squares mean (±SE) numbers of various instars from *S.* *pyrioides* in (**A**) the first, second, third and fourth, and fifth instars and (**B**) the total nymphs and exuviae at 7 d posttreatment when IGRs were applied to implanted *S. pyrioides* eggs. The bars of the same fill color with the same letters are not significantly different (pairwise *t-*test, *p* = 0.05). Nonsignificant data have no letters.

The numbers of first instars were not significantly different between treatments (*F*_4, 36_ = 1.1; *p* = 0.388; Figure 1A). The numbers of second instars were significantly lower for novaluron, azadirachtin, and buprofezin than for pyriproxyfen and the nontreated control treatments (*F*_4, 36_ = 9.3; *p* < 0.001). Similarly, the densities of third and fourth instars were significantly lower under novaluron, azadirachtin, and buprofezin than pyriproxyfen and the nontreated control treatments (*F*_4, 36_ = 4.2; *p* = 0.006). There were no significant differences in the numbers of second and third instars between the novaluron, azadirachtin, and buprofezin treatments. For the fifth instars, there was no significant difference between treatments (*F*_4, 36_ = 1.4; *p* = 0.311). 

When all the nymphs were combined, the number of total nymphs was significantly lower in response to the novaluron, azadirachtin, and buprofezin than the pyriproxyfen and nontreated control treatments (*F*_4, 75_ = 7.5; *p* < 0.001; Figure 1B). There was no significant difference in nymphal densities between the novaluron, azadirachtin, and buprofezin treatments. The numbers of exuviae shed by the nymphs were significantly lower in the azadirachtin than in the novaluron, pyriproxyfen, and buprofezin treatments followed by the nontreated control (*F*_4, 75_ = 11.9; *p* < 0.001).

### 3.2. Translaminar Effects on Implanted Eggs and Nymphs

**Figure 2:**
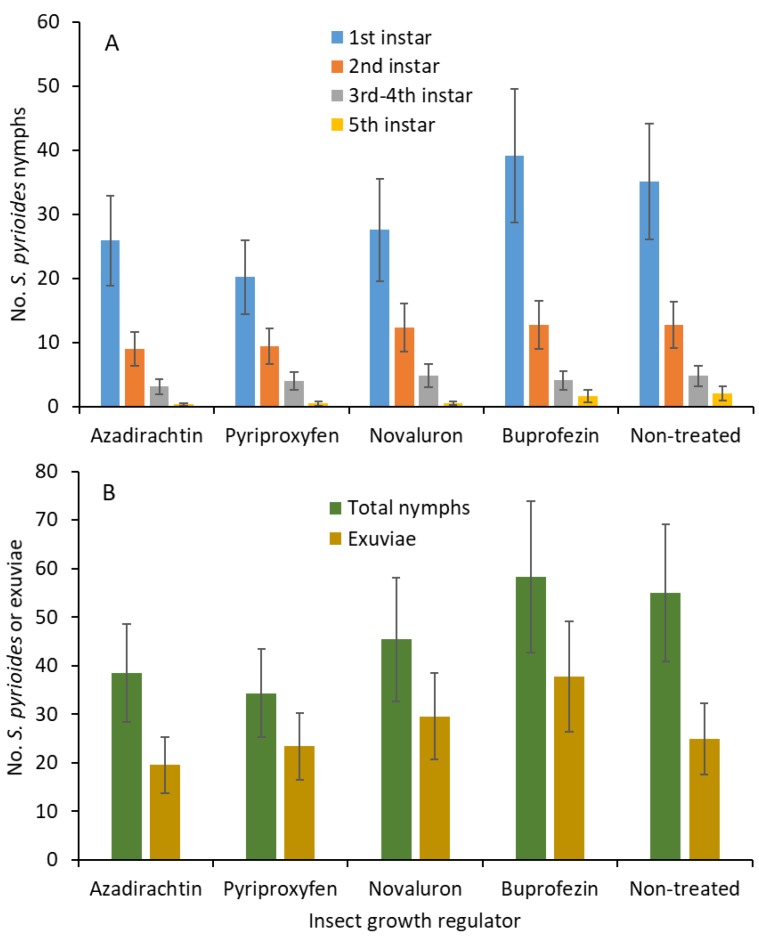
Least squares mean (±SE) numbers of various instars from *S. pyrioides* in (**A**) the first, second, third and fourth, and fifth instars and (**B**) the total nymphs and exuviae at 7 d posttreatment when IGRs without adjuvant were painted onto the adaxial surfaces of the leaves after *S. pyrioides* eggs were implanted. Bars of the same fill color with the same letters are not significantly different (pairwise *t-*test, *p* = 0.05). Nonsignificant data have no letters.

When adjuvant was not added, there were no significant differences for the first (*F*_4, 54_ = 0.8; *p* = 0.496; Figure 2A), second (*F*_4, 54_ = 0.4; *p* = 0.812), third and fourth (*F*_4, 54_ = 0.2; *p* = 0.860), and fifth (*F*_4, 54_ = 1.9; *p* = 0.119) instars between IGR treatments. When all the instars were combined, the nymphal densities (*F*_4, 54_ = 0.8; *p* = 0.553; Figure 2B) and shed exuviae (*F*_4, 54_ = 0.9; *p* = 0.461) were not significantly different between treatments.

**Figure 3:**
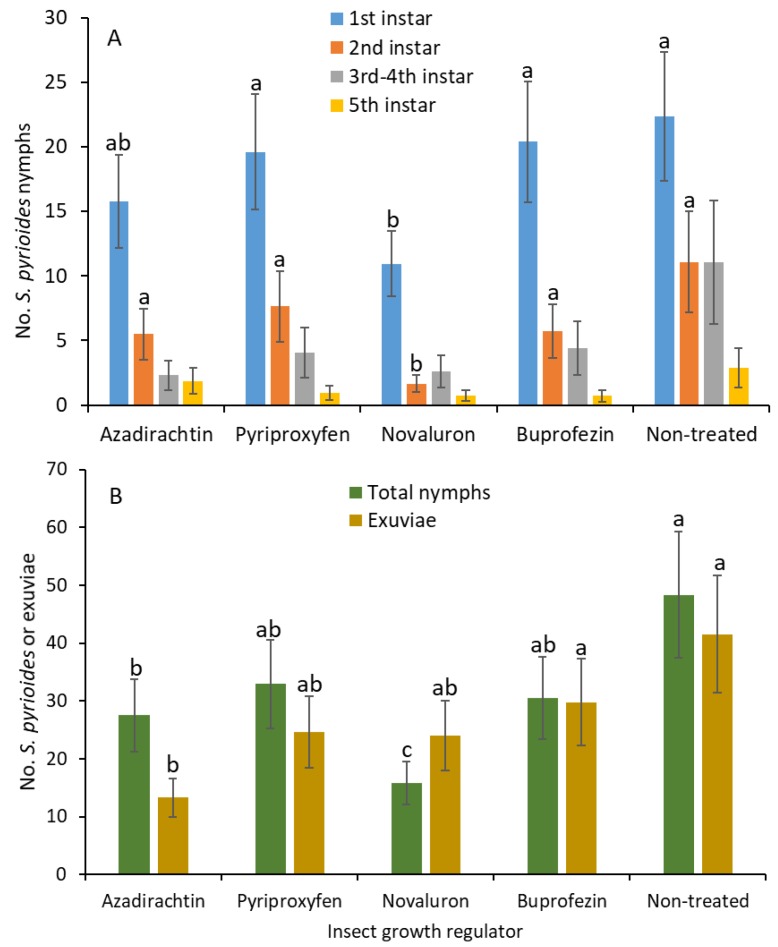
Least squares mean (±SE) numbers of various instars from *S. pyrioides* in (**A**) the first, second, third and fourth, and fifth instars and (**B**) the total nymphs and exuviae at 7 d posttreatment when IGRs with adjuvant were painted on the adaxial surfaces of leaves after *S. pyrioides* eggs were implanted. Bars of the same fill color with the same letters are not significantly different (pairwise *t*-test, *p* = 0.05). Nonsignificant data have no letters.

When adjuvant was added, the number of first instars was significantly lower in the novaluron than in the buprofezin, pyriproxyfen, and nontreated control treatments (*F*_4, 54_ = 2.6; *p* = 0.049; Figure 3A). There was no significant difference in the number of first instars between the novaluron and azadirachtin treatments. Similarly, the second instars were significantly lower in the novaluron treatment than in the azadirachtin, buprofezin, pyriproxyfen, and nontreated control treatments (*F*_4, 54_ = 6.3; *p* < 0.001). There were no significant differences for the third and fourth (*F*_4, 54_ = 1.9; *p* = 0.109) and the fifth instars (*F*_4, 54_ = 1.2; *p* = 0.306) between treatments. The densities of total nymphs were significantly lower in the novaluron and azadirachtin than in the buprofezin, pyriproxyfen and nontreated control treatments (*F*_4, 54_ = 5.7; *p* < 0.001; Figure 3B). In addition, between novaluron and azadirachtin, the numbers of nymphs were significantly lower in the novaluron than in the azadirachtin treatment. There was no significant difference between the buprofezin, pyriproxyfen, and nontreated control treatments for the newly emerged nymphal densities. The numbers of shed exuviae were significantly lower under azadirachtin than in the nontreated control (*F*_4, 54_ = 3.6; *p* = 0.012). There were no significant differences between azadirachtin and buprofezin, pyriproxyfen, or novaluron on the exuviae densities.

### 3.3. Assessing Ovicidal Effects

**Figure 4:**
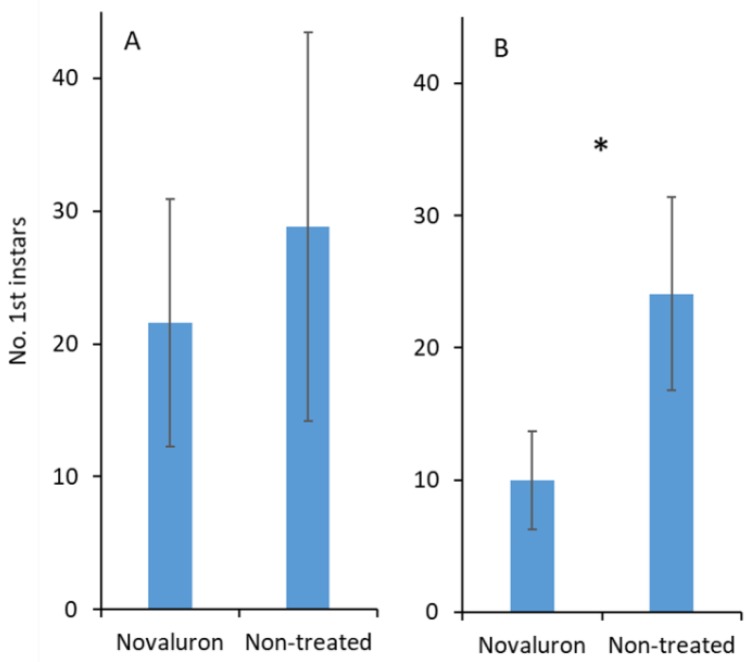
Mean (±SE) numbers of first instars from *S. pyrioides* that emerged from the eggs that were inserted into novaluron-treated leaves and the nontreated control (**A**) without and (**B**) with adjuvant added. Pairs of bars with asterisks (*) indicate significantly differences at *α* = 0.05 (Student’s *t*-test).

When adjuvant was not added, the number of first instars that emerged from the eggs was not significantly different between the novaluron and nontreated treatments (*t*_18_ = 1.8; *p* = 0.091; Figure 4A). There was a significant difference in egg hatching when an adjuvant was added, and there was a greater number of hatched eggs in the nontreated control than in the novaluron treatment (*t*_25_ = 2.4; *p* = 0.022; Figure 4B).

### 3.4. Direct Exposure of First Instars

**Figure 5:**
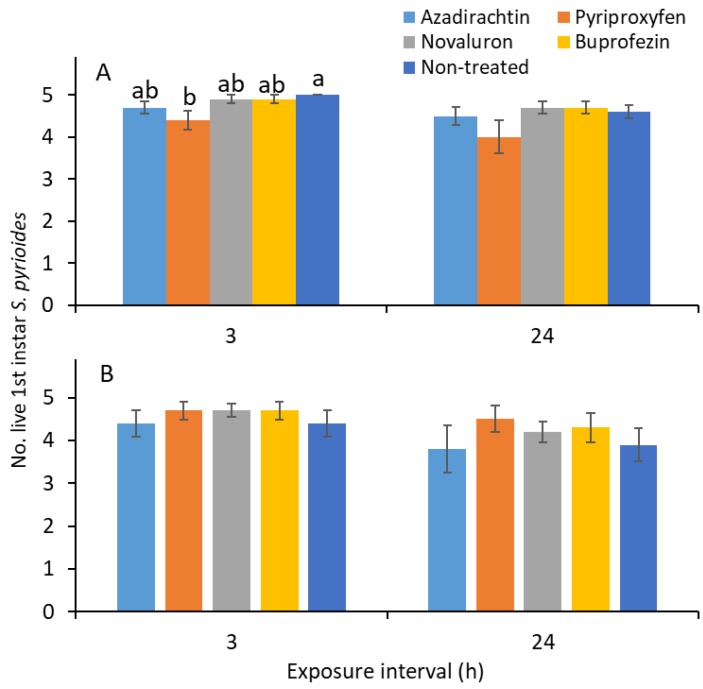
Mean (±SE) numbers of first *S. pyrioides* instars that survived after 3 and 24 h of exposure to aged IGR residues for (**A**) 1 and (**B**) 7 d after spray application. Bars with the same letters within the exposure time are not significantly different at α = 0.05 (Tukey’s HSD test). Nonsignificant data have no letters.

For 1 d-old residue, the results show that the numbers of live first instars were significantly different for various IGR treatments (*F*_4, 81_ = 3.8; *p* = 0.007) and exposure times (*F*_1, 81_ = 5.4; *p* = 0.022); however, the IGR treatment × exposure time interaction was not significantly different (*F*_4, 81_ = 0.2; *p* = 0.954). When the direct effects of IGRs were examined, the number of live nymphs was significantly lower in the pyriproxyfen than the nontreated control treatment after 3 h of exposure (*F*_4, 36_ = 3.2; *p* = 0.026; Figure 5A). There was no significant difference between IGR treatments in terms of the number on first instars after 24 h of exposure (*F*_4, 36_ = 1.4; *p* = 0.244; Figure 5A).

For the 7 d-old residue, the exposure time effect was significantly different in the surviving first instars (*F*_1, 81_ = 6.8; *p* = 0.011). The other factors, namely, the IGR treatment (*F*_4, 81_ = 1.4; *p* = 0.244) and IGR treatment × exposure time interaction (*F*_4, 81_ = 0.2; *p* = 0.957), were not significantly different among the live nymphs. When examined specifically for the exposure intervals of 3 (*F*_4, 36_ = 0.5; *p* = 0.709; Figure 5B) and 24 h (*F*_4, 36_ = 0.9; *p* = 0.478), the surviving nymph results were not significantly different between IGR treatments. 

## 4. Discussion

The results show that the IGRs novaluron, buprofezin, and azadirachtin reduced the number of *S. pyrioides* nymphs when applied to leaves containing implanted *S. pyrioides* eggs. The data suggest that this IGR activity against nymphs was neither related to the direct contact toxicity in newly emerged first instars nor the ovicidal effects. Signs of ovicidal activity from novaluron were evident only when an adjuvant was added to the novaluron. *Stephanitis pyrioides* overwinters in the form of eggs in the eastern USA, and 50% of those overwintering eggs hatched in March, when 211 degree days had accumulated [10]. Thereafter, the insects passed four generations in Georgia [10]. Any ovicidal or nymphicidal effects on the overwintering eggs or emerging young instars can effectively suppress the population build-up later in the season. Previous studies showed that IGRs target the developing immature stages of several insect pests, such as azadirachtin, on *Corythucha ciliata* (Say) (Hemiptera: Tingidae) [25]; novaluron and methoprene on the storage pests *Tribolium castaneum* and *T. confusum* (Coleoptera: Tenebrionidae) [26]; novaluron and diflubenzuron on *Bagrada hilaris* (Burmeister) (Hemiptera: Pentatomidae) [27]; and pyriproxyfen on the sweet potato whitefly *Bemisia tabaci* (Gennadius) and the greenhouse whitefly *Trialeurodes vaporariorum* (Westwood) [19].

No ovicidal activity was evident against *S. pyrioides* when only IGRs were sprayed on the leaves containing implanted eggs (Figure 2). Any evidence of ovicidal activity also suggests that the IGRs can affect the developing nymphs [13]. It is not clear why the IGRs did not elicit ovicidal activity. One reason could be related to the oviposition behavior of the *S. pyrioides* females. Females choose to insert their eggs mostly into the base of either side of the midrib, on the abaxial surface of the azalea leaf. This region of the leaf is the thickest, and the eggs are completely embedded and protected. Once the eggs are inserted into the leaf tissue, the females cover the eggs with a layer of black cement-like fecal matter on the opercula, which probably armors the implanted eggs from external elements such as insecticide exposure, predation, or parasitization. This oviposition behavior is likely to play a major part by reducing exposure to the applied insecticide residues, including those from the IGRs. Similarly, not all previous studies using IGRs have reported adequate ovicidal activity against other hemipteran pests, for example, azadirachtin on the rice bug *Leptocorisa chinensis* (Dallas) [28] and pyriproxyfen on the Asian citrus psyllid *Diaphorina citri* [29]. Another reason is a lack of translaminar activity for IGRs. There are limited studies showing the translaminar activity of IGRs.

The results show the novaluron elicited translaminar activity against *S. pyrioides* when an adjuvant was added, and fewer eggs hatched and nymphs survived compared to the nontreated control (Figure 3 and Figure 4). *Stephanitis pyrioides* colonizes the abaxial side of the leaf and feeds on the chlorophyll in the upper parenchyma cells by inserting its stylets through the stomatal opening [1]. This feeding and colonizing behavior also suggests that having systemic and/or translaminar activity will assist in effective *S. pyrioides* control. Azalea bushes in ornamental landscapes and gardens usually have a dense canopy. Achieving proper penetration of insecticide material through dense canopies and specifically reaching the abaxial surfaces of the leaves can be challenging. When insecticides are sprayed, the insecticide residues are usually deposited on the adaxial surfaces of the azalea leaves. In the current study, although the results show that IGRs did not elicit any translaminar activity against *S. pyrioides* eggs or nymphs in the absence of adjuvant, in the presence of an adjuvant, applying novaluron to the adaxial surface of the leaves reduced egg hatching and the number of young nymphs on the abaxial surface of the leaves, indicating signs of translaminar activity. Specifically, evidence of the lower density of first instars in the novaluron plus adjuvant treatment suggest that the translaminar movement of novaluron residues is likely affecting *S. pyrioides* egg hatching. Thus, these data suggest that IGRs, especially novaluron, can suppress the egg hatching and development of young *S. pyrioides* instars when used with an adjuvant. Similarly, translaminar activity was reported for other piercing and sucking insect pests such as the sweet potato whitefly *Bemisia tabaci* (Gennadius) and the greenhouse whitefly *Trialeurodes vaporariorum* (Westwood) when pyriproxyfen was applied to the adaxial surface of the cotton leaves [19]. However, studies supporting the enhanced translaminar activity of IGRs with added adjuvant against insect pests are limited.

The results show that exposing young instars to IGR residues on the leaf surface did not directly lead to mortality (Figure 5). This finding indicates that the reduced numbers of second as well as third and fourth instars observed in novaluron, buprofezin, and azadirachtin treatments (Figure 1) were mostly related to mortality that occurred during the molting process. A previous study [12] showed that novaluron and buprofezin reduced *S. pyrioides* egg hatching when the adults were exposed to the dried IGR residues. Perhaps the dried residues of novaluron and buprofezin on the foliage could impact the newly emerged adults, which could suppress population development [12], although novaluron and buprofezin have no effect on the survival of the adults. 

## 5. Conclusions

The data show that novaluron, buprofezin, and azadirachtin reduced the development of young nymphs when *S. pyrioides* eggs implanted in leaves were exposed to IGRs. When the adaxial surface of the leaves was treated with IGRs alone, no ovicidal or nymphicidal effects were noted; however, novaluron reduced the numbers of eggs that hatched and first instars when an adjuvant was added. This suggests that ovicidal and nymphicidal properties of IGRs especially, novaluron against *S. pyrioides* can be enhanced by adding an adjuvant. These results have implications for *S. pyrioides* management because the use of neonicotinoids has been discontinued or dramatically reduced by nursery and professional landscape mangers in the USA. In addition, overdependence on pyrethroid insecticides can be reduced through the adoption of IGRs as a management option because pyrethroid insecticides will affect the survival of nontargets, including beneficial arthropods, such as predators and parasitoids, which pose a serious risk of secondary pest outbreak.
